# Use of Public Oral Health Services by the Adult Population: A Multilevel Analysis

**DOI:** 10.1371/journal.pone.0145149

**Published:** 2016-01-05

**Authors:** Rafaela da Silveira Pinto, Angelo Giuseppe Roncalli, Mauro Henrique Nogueira Guimarães Abreu, Andréa Maria Duarte Vargas

**Affiliations:** 1 Oral Health Department of Minas Gerais, Minas Gerais State Health Department, Belo Horizonte, Minas Gerais, Brazil; 2 Department of Community and Preventive Dentistry, School of Dentistry, Universidade Federal de Minas Gerais, Brazil., Belo Horizonte, Minas Gerais, Brazil; 3 Department of Dentistry, Federal University of Rio Grande do Norte, Natal, Rio Grande do Norte, Brazil; Columbia University, UNITED STATES

## Abstract

**Background:**

It is important to assess context to explain inequalities in oral health, particularly with regard to the type of service used; thus, this study aimed to identify the social determinants of public dental service use by adults and to assess whether, beyond the level individual, existing inequalities are also expressed in the context in which individuals are embedded.

**Methods:**

A multilevel analysis with three levels of aggregation of variables was performed. The individual variables were derived from the database of the SB Minas Gerais project—a survey of oral health status of the population of Minas Gerais, a state of the Brazilian Southeast region. The variable at the neighborhood level came from the Census of 2010. The variables at the municipal level were obtained from available public databases relating to oral health services. At the municipal level, the Human Development Index (HDI) variable was chosen to represent quality of life in the municipalities.

**Results:**

In the final model, the following individual variables were associated with greater use of public dental services: lower income (PR = 1.98, 95% CI = 1.53; 2.58), higher number of residents at home (PR = 1.37, 95% CI = 1.11; 1.68) and higher number of teeth requiring treatment (PR = 1.49, 95% CI = 1.20; 1.84). With regard to context variables, a poorer infrastructure (PR = 0.62, 95% CI = 0.40; 0.96) leads to a lower use of public services.

**Conclusion:**

The use of public services is associated with family income, how this income is divided in households, the need for treatment presented by the individual and the organization of the existing oral health service infrastructure in the municipality.

## Introduction

Oral care is the third most prevalent cause for health care demand in Brazil [1]; however, there are inequalities in the use of these services.

Several studies have addressed the use of oral health services for populations in various locations in the world [2–11]. In the Brazilian context, the following areas have been investigated: the use of health services in general [12–14], the use of dental care for the elderly [15–17], the regular use of dental services [15, 18–20], the use of dental services motivated by pain [21], the use of secondary care in oral health [22,23] and the characterization of the use of dental services [24–26]. Only three Brazilian studies were identified that investigated the type of service as an outcome of interest in the adult population [27–29].

Oral public health services must have an adequate infrastructure to meet the population's needs. Thus, knowing the factors associated with the use of public dental services can provide necessary information about the reasons that lead individuals to use such services and to the formulation of public policies suited to reality.

Considering the importance of assessing the context in which individuals live to explain inequalities in oral health, particularly with regard to the type of service used, this study aimed to identify the social determinants of public dental service use for adults. In addition to assess whether, beyond the individual level, inequalities are also expressed in the context in which individuals are embedded.

## Materials and Methods

### Studied Area

The Minas Gerais state is the second largest Brazilian state based on the number of inhabitants; it has the largest number of municipalities (853) and is situated in the southeast area of the country [30]. When the epidemiological investigation was conducted to collect the individual variables in this study (2012), there were 14,252 dentists in the state, and 49.5% of these dentists were working in the public health system [31] at a ratio of 1 dentist in public service for every 2,817 inhabitants on average. In Minas Gerais, 26.3% of residents possessed private health insurance coverage, and only 7.1% were insured by dental plans [32].

### Study Variables

#### Dependent Variable

The participants of the SB Minas Gerais Project [33] were asked whether they had ever been to the dentist at least once in their life, and if so, what type of service was used. Those who responded positively to the first question were eligible for analysis in this study.

The dependent variable was the type of service used in the last dental appointment, which could be characterized as public, private, health insurance and others. The original variable was dichotomized into public service and private service (which includes private care, health insurance and others).

#### Municipal Level Variables

The data on health services in the municipalities of Minas Gerais in 2012 were extracted from the public health information system of the Ministry of Health, the DATASUS system [34], the Management Support Center (MSC) [35] and the Supplementary National Health Agency (SNHA) [32]. Initially, data were collected for thirty-six variables related to oral health services. Nine variables were selected to create the characterization factors of these services in the state (Table 1) that had similar dimensions and significant correlations between them. A factor analysis was performed based on Principal Components Analysis (PCA) with standardized scores of the nine original variables. Data considered outliers (greater than three standard deviations) were replaced by the mean [36].

**Table 1:** Independent variables according to the level of analysis.

Level	Variable		Description	Source of data
**1**^**st**^ **Level—Individual**	Race/Skin Color		Self-reported skin color. From the five original categories, a dichotomous variable was created (white and non-white).	SB Minas Gerais
	Number of people living at home		Number of people, including the respondent, residing at home.	SB Minas Gerais
	Family Income		Total income received by all family members in the month preceding the survey, in Brazilian Reais.	SB Minas Gerais
	Number of teeth requiring treatment		Total number of teeth requiring treatment for caries	SB Minas Gerais
**2**^**nd**^ **Level—Neighborhood (Census Sector)**	Factor 1—Neighborhood conditions	Adequate household	Adequate household: piped water, sewage collection, garbage collection and no more than two persons per bedroom.	Census 2010
		Paved streets	% of households with available paved streets	Census 2010
		Sidewalk	% of households with available sidewalk	Census 2010
		Curb	% of households with available curb	Census 2010
	Factor 2—Socioeconomic status of households	Average number of residents	Average number of household residents	Census 2010
		Average household income	The average monthly nominal yield value of people 10 years or older (with and without income)	Census 2010
		Households with up to 1 minimum wage	% of households with monthly nominal income per capita of up to 1 minimum wage	Census 2010
		Non-white residents	% non-white residents	Census 2010
		Literate sponsors	% literate sponsors	Census 2010
**3**^**rd**^ **Level—Municipalities**	HDI		Human Development Index	Atlas PNUD
	Factor 1—Services: Infrastructure	Oral health coverage in primary health care	Oral health coverage in primary health care (conventional and family health program)	DATASUS
		Dental plan coverage	Population coverage of dental plan	HNA
		Proportion of dentists in public service	Proportion of dentists in public service	DATASUS
		Dentists in the private service	Number of dentists working in the private service per 10,000 inhabitants	DATASUS
		Oral health resources in primary health care	Oral health resources in primary health care *per capita*	MSC
	Factor 2—Services: Dental procedures	Proportion of tooth extractions	Proportion of extraction in relation to individual basic dental procedures	DATASUS
		Average tooth brushing	Average supervised tooth brushing	DATASUS
		Specialized procedures	Number of specialized oral health procedures per 10,000 inhabitants	DATASUS
	Factor 3—Services: Dental Prostheses	Prostheses	Number of dental prostheses per 10,000 inhabitants	DATASUS

In addition to the oral health service variables that composed the factor analysis, the Human Development Index (HDI) [37] was also selected to compose the group of variables at the municipal level because it is a composite indicator that includes educational levels, longevity and income and is generally used as a synthetic indicator for the level of quality of life of populations [38].

#### Neighborhood Level Variables—census sector

In Brazil, the census sectors have an average of 300 households and similar socioeconomic characteristics [39].

For this study, data from the Minas Gerais State urban census sector were extracted from the National Demographic Census that was conducted in 2010 [40]. Initially, thirty variables were selected. Considering the large number of variables, a preliminary correlation analysis was performed, and nine variables were chosen. The selected variables are presented in Table 1. A PCA was then performed with the nine variables at this level.

#### Individual Level Variables

The individual level variables were obtained from the database of the SB Minas Gerais project, which was a cross-sectional epidemiological survey of oral health status that was conducted on the population of Minas Gerais in 2012 [33]. This study investigated the main oral health diseases at the ages of 5 and 12 and in the age groups 15–19, 35–44 and 65–74; related socio-economic aspects were also evaluated. The methodological basis was the same as the national survey that was conducted in Brazil in 2010 [41], both with regard to the collected indices, team training, sampling methodology and the fieldwork (collection route). The sample size was also based on the severity of dental caries estimated by the DMFT (number of teeth decayed, missing and filled), according to data from the SB Brazil 2010 for the Southeast region. For each age group and each domain, the prevalence of caries and the average DMFT were used as a reference for the sample size calculation associated with an error rate. In the age group of 35–44, this error was estimated as 5% [33]. The proposed design ensures the production of inferences to estimate the caries attack to the state of Minas Gerais and for each domain, considering each age or age group. For other diseases, the representability degree varied with the estimated prevalence and severity. The overall response rate was 81.1%, slightly above and was therefore within the parameter established in the sample plan (80%). The details of the sampling plan for the database are available in the final report of the project [33]. The survey included a representative sample for the state of Minas Gerais and three areas (capital and two non-capital domains). The sample calculation subsequently revealed that the sample studied assured a confidence level of 95% and 80% power for the variables used in this study [28].

The choice of individual variables in this study was based on the work of Pinto *et al*. (2014) [28] that used the same database for the investigation of individual characteristics that lead adults to use public oral health services. These include: race/skin color (white, non-white), number of people living at home (1–4 people, 5 or more people), family income (greater than 1,501 Brazilian Reais, up to 1,500 Brazilian Reais—at the time of the SB Minas Gerais project, USD1 = 2 Brazilian Reais), and number of teeth requiring treatment (up to 1.72 teeth, 1.73 and more teeth).

### Data Analysis

First, PCA was performed for the municipal level context variables (except HDI) and the neighborhood variables, as described above. After, an exploratory analysis assessing the effect of each variable in the outcome was performed, calculating the prevalence ratios (PRs) and their respective confidence intervals using the best situation as the reference category. Those variables with p <0.20 were included in the multilevel model, as described below.

A three-level multilevel mixed-effects Poisson regression analysis was performed to verify the individual characteristics and the influence of context on the outcome. In this study, the context was represented by two levels of aggregation, census sectors and cities, taking into account the administrative organization of the state of Minas Gerais. Individuals were grouped into census sectors, which, as described above, represent similar socioeconomic characteristics. This level represents the contextual effect of neighborhood. Finally, the census sectors are nested in cities, the third level. Thus, the database that was analyzed contains data from 236 census sectors and 57 cities selected for research in the SB Minas Gerais.

The multilevel analysis was performed in four steps. First, the null model was estimated without variables, only splitting the variance in the three levels of analysis. The variables were inserted into the null model in blocks according to the levels listed above. For the following models, prevalence ratios and their confidence intervals were estimated. "Model 1" included only the variables at the individual level. "Model 2" included all variables with concomitant adjustment of the individual variables that had p <0.20 and were contextual at the neighborhood level (census sector). The "final model" included all factors with concomitant adjustment of the individual and contextual variables at the municipal level (because the variables of the neighborhood level were not significant when inserted into the model). Changes in the quality adjustment models were analyzed by the likelihood ratio test. PRs were estimated (95% CI) for each variable [42].

### Ethical Clearance

Ethical clearance was not required because the data obtained from the Brazilian National Health Information System of the Ministry of Health were public, aggregated and anonymous. Written consent was not obtained because the data were public and aggregated at the municipal level. Thus, patient information was anonymized and de-identified prior to analysis. The individual data came from the public database of the SB Minas Gerais Research that was approved by the Ethics Committee of the Pontifical Catholic University of Minas Gerais in the document n° 9173 of 28 March 2012. Patient information was anonymized and de-identified prior to analysis.

## Results

The variables at the municipal level and neighborhood level were evaluated based on the correlation between these variables. All Pearson’s correlation values were significant and were between 0.30 and 0.90.

The PCA of the municipal level identified 3 factors that explained 56% of the total variance. After being rotated by the Varimax method, the eigenvalues were calculated and are presented in Table 2. The value for the Kaiser-Meyer-Olkin (KMO) was 0.662, which was considered reasonable, and Bartlett's test of sphericity was significant (p <0.001). The factors were then called: factor 1—"infrastructure", factor 2—"dental procedures" and factor 3—" dental prostheses".

**Table 2:** Rotated component matrix for the variables included in the factor analysis for municipalities—oral health services.

Municipalities—Dental Services	Component*		
Variable	1Infrastructure	2Dental Procedures	3Dental Prostheses
Dental plan coverage	-0.625		
Oral health coverage in primary health care	0.721		
Oral health resources in primary health care	0.731		
Dentists in the private service	-0.746		
Proportion of dentists in public service	0.832		
Specialized procedures		0.342	
Proportion of tooth extractions		-0.751	
Average tooth brushing		0.599	
Dental prostheses			0.700

*Principal Component Analysis was the extraction method, and the rotation was Varimax with Kayser normalization.

In the case of the PCA at the neighborhood level, two factors were created to represent the original variables that explained 71% of the total variance. After being rotated by the Varimax method, the eigenvalues were calculated and are presented in Table 3. The value for the Kaiser-Meyer-Olkin (KMO) was 0.841, indicating a good fit of the sample, and the Bartlett's test of sphericity was significant (p <0.001). Considering each variable group formed, factor 1 was called "neighborhood conditions" and factor 2 was called "socioeconomic status of households".

**Table 3:** Rotated component matrix for the variables included in the factor analysis for census sector.

Census Sector	Component*	
Variable	1Neighborhood conditions	2Socioeconomic status ofhouseholds
Paved streets	0.908	
Kerb	0.903	
Sidewalk	0.819	
Adequate household	0.758	
Literate sponsors		-0.613
Households with up to 1 minimum wage		0.871
Non-white residents		0.775
Average number of residents		0.716
Average household income		-0.835

*Principal Component Analysis was the extraction method, and the rotation was Varimax with Kayser normalization.

At the individual level, the records of 1,110 adults 35–44 years old who had been to the dentist at least once in their life were obtained from the database of the epidemiological survey of SB Minas Gerais. There were missing data for the variables income (1.54%) and the number of teeth requiring treatment (0.18%). Because none of the variables had more than a 2% loss, data imputation was not performed. For the multilevel analysis, the records with complete data were used (n = 1,091 individuals).

Table 4 presents the descriptive analysis of the three levels of this study (individual, neighborhood and municipal) and the bivariate analyses of the outcome public service use. This table shows that for the individual variables, women, people with lower incomes and people with more teeth in need of treatment use more public services than private services. It is noteworthy the importance of income: people with lower incomes use public service twice as often compared to private service when analyzed in isolation from a bivariate perpective.

**Table 4:** Bivariate associations between outcome and the independent variables according to the levels.

Variable		Use of public service		
		n	PR	p-value
**Individual Level**				
Race/skin Color	White	131	Ref	0.012
	Non-white	255	1.33 (1.06;1.65)	
Number of people living at home	1–4 people	226	Ref	p<0.001
	5 and more people	160	1.45 (1.18;1.78)	
Family Income	1,501 Brazilian Reais or more	87	Ref	p<0.001
	Up to 1,500 Brazilian Reais	295	2.29 (1.78;2.96)	
Teeth requiring treatment	Up to 1.72 teeth	195	Ref	p<0.001
	1.73 or more teeth	190	1.68 (1.37;2.07)	
**Neighborhood Level**				
Factor 1—Neighborhood conditions	0.145 and more	182	Ref	0.167
	Up to 0.145 (worst conditions)	204	1.19 (0.93;1.52)	
Factor 2—Socioeconomic status of households	Up to 0.426	138	Ref	0.011
	0.426 and more (worst conditions)	248	1.36 (1.07;1.72)	
**Municipal Level**				
HDI	0.697 and more	206	Ref	0.009
	Up to 0.697 (worst HDI)	180	1.50 (1.11;2.03)	
Factor 1—services: Infrastructure	-0.247 and more	180	Ref	p<0.001
	Up to -0.247 (worst infrastructure)	206	0.59 (0.44;0.79)	
Factor 2—services: Dental procedures	-0.139 and more	148	Ref	0.709
	Up to -0.139 (worst offer)	238	1.06 (0.77;1.46)	
Factor 3—services: Dental prostheses	-0.035 and more	233	Ref	0.962
	Up to -0.035 (worst offer)	153	0.99 (0.72;1.36)	

In relation to the neighborhood level, conditions of the worst surrounding and socioeconomic characteristics of the households were positively associated with the use of public services (Table 4). At the municipal level, those with the worst HDI, which represents quality of life in cities, better infrastructure of health services and lower supply of prostheses, were positively associated with the outcome. The offer of individual procedures and prevention (factor 2—services: dental procedures) was not statistically significant for inclusion in the multilevel model according to the established criteria (p <0.20).

Table 5 shows the null model in which both the neighborhood level and the municipal level have statistical significance for fixed and random effects.

**Table 5:** Fixed and random effects parameters in the multilevel mixed-effect Poisson regression analysis for the null model according to outcome.

Null Model			
		**Fixed Effects**	
		**Intercept**	**95% CI**
Municipal Level		-1.07	(-1.24;-0.91)
Neighborhood Level		-1.10	(-1.22;-0.98)
Both		-1.07	(-1.24;-0.91)
		**Random Effects**	
		**Variance (SE)**	**LR Test (Chi-Square test; p)**
Municipal Level only		0.169 (0.066)	30.14; <0.001
Neighborhood Level only		0.102 (0.053)	5.80; 0.008
Both	0.169 (0,065)	30.14; <0.001	30.14; <0.001
	Neighborhood Level	0.000 (0.000)	

CI = Confidence Interval;

LR = Likelihood Ratio;

SE = Standard Error.

When individual variables were included in "model 1" (Table 6), the outcome prevalence was significantly associated with the number of residents in the household, income and the number of teeth requiring treatment. However, race/skin color lost its significance. The variables for the neighborhood level, when inserted into “model 2”, showed no statistical significance and hence were removed in the next step. In the "final model", the municipal variables were included, but only the factor related to the infrastructure of services remained significant.

**Table 6:** Multilevel mixed-effect Poisson regression analysis for the outcome.

	Model 1 (n = 1,091)		Model 2 (n = 1,091)		Final Model (n = 1,091)	
**Municipal Level**	**PR (95% CI)**	**p-value**	**PR (95% CI)**	**p-value**	**PR (95% CI)**	**p-value**
HDI (≤0.697)					0.91 (0.58;1.42)	0.676
Factor 1—services: Infrastructure (worst infrastructure)					0.62 (0.40;0.96)	0.033
**Neighborhood Level**	**PR (95% CI)**	**p-value**	**PR (95% CI)**	**p-value**	**PR (95% CI)**	**p-value**
Factor 1—Neighborhood conditions (worst conditions)			1.08 (0.85;1.37)	0.555	-	-
Factor 2—Socioeconomic status of households (worst conditions)			1.07 (0.83;1.37)	0.611	-	-
**Individual Level**	**PR (95% CI)**	**p-value**	**PR (95% CI)**	**p-value**	**PR (95% CI)**	**p-value**
Race/skin Color (Non-white)	1.16 (0.93;1.45)	0.202	-	-	-	-
Number of people living at home (5 or more people)	1.35 (1.09;1.66)	0.005	1.36 (1.10;1.67)	0.004	1.37 (1.11;1.68)	0.003
Family Income (≤ 1,500 Reais)	2.08 (1.61;2.70)	0.001	2.05 (1.57;2.67)	<0.001	1.98 (1.53;2.58)	<0.001
Number of teeth requiring treatment (1.73 or more teeth)	1.43 (1.16;1.78)	<0.001	1.46 (1.18;1.80)	0.001	1.49 (1.20;1.84)	<0.001
**Fixed effects**	**PR (95% CI)**	**p-value**	**PR (95% CI)**	**p-value**	**PR (95% CI)**	**p-value**
Intercept (95% CI)	-1.91 (-2.21;-1.61)	<0.001	-1.89 (-2.19;-1.60)	<0.001	-1.50 (-1.99;-1.00)	<0.001
**Random effects**	**Variance (SE)**		**Variance (SE)**		**Variance (SE)**	
Municipal Level	0.128 (0.054)		0.112 (0.051)		0.094 (0.044)	
Neighborhood Level	0.000 (0.000)		0.000 (0.000)		-	
LR Test (Chi-Square Test; p-value)	24.67 (<0.001)		17.99 (<0.001)		12.70 (<0.001)	

The comparison between the setting of quality measurements for the null model, the model with the individual level (model 1) and the full multilevel model (late model) indicates that at each stage of the analysis, there was a significant gain of explanation of variance of the outcome. Thus, the difference of these variances between the null model and the final model was 44.37%.

## Discussion

In this study, the individual variables of lower family income, greater number of household residents and more teeth requiring treatment were associated with the outcome. In the municipal context, better infrastructure leads to a greater use of public services. In recent years, a decrease in tooth decay prevalence has been identified, but inequalities remain in this scenario [43], and the challenges faced by public health policies are still great [44]. The complexity of the causes of health inequalities expresses the need for multisectoral actions in public health to address the negative macro-environmental factors and the physical and social environment. In addition, action is required regarding adverse health behaviors and access to health care [45]. In this sense, some authors propose that studies such as this one, which integrate individual and contextual determinants to explain the use of public services, should be used as a strategy to reduce health inequalities [44].

In this study, we found that having many residents in a household increases the prevalence of public service use. One possible explanation is that the increase in the number of residents in a home indicates a greater number of individuals who are dependent on the family income and therefore have a reduced ability to afford private services. Thus, the decision of which service to use would be based on the complete needs of all the people who depend on the same family income and not just those of the service user.

In a study of elderly people in southern Brazil, individuals belonging to larger families used less private health services, and the addition of a family member decreased the chances of older people using private care by 15% [46]. Regardless of the type of service studied, associations in both directions between the size of families and the use of services have been found in the literature. The findings have been inconsistent because the association between the variables can be influenced by the current health system [47,48].

The inclusion of individuals within families should be considered an important factor in the construction of public policies. Although the interactions between household residents represent social support, these interactions also point to restrictions of the use of financial resources on the demand for care when the number of residents increases [46] in our study, particularly in public service.

One obstacle to the use of oral health services is the financial cost of dental care and its relationship to income and the different health systems [9], both in developed and developing countries. Income has been identified as an explanatory factor for the use of oral health services [4, 6, 8–11, 49, 50] in several countries with different health systems. In Brazil, although there is a universal health care system [51], similar barriers are observed relating to income and therefore to the cost of services [15–20, 27, 52, 46, 53, 54].

It is crucial to consider the variation in the format of existing health systems in each country, given its contribution to the complexity of the analysis. Often the differences found in the permanence of some explanatory factors result from the formatting of the available network services [48].

The association for the increased use of public service among people with more teeth requiring treatment is consistent with the literature [55] because these individuals are generally those with a low socioeconomic status. The need for tooth cleaning is a variable of adjustment to the other individual variables.

The Brazilian National Health System (SUS, from Portuguese acronym) plays a key role in reducing inequalities in health and in the provision of universal access to care. In this study, we found that municipalities that have better-structured public services networks lead a more frequent use of these services by adults.

However, the limitations of the public health system in solving people's problems must be considered. For the oral health care network to be able to respond to the health needs of the population, the way in which people obtain access must be strengthened. Access should be facilitated with a range of geographically well-distributed services. Moreover, it should find efficient mechanisms to regulate the flow of assistance to other levels of care to achieve integrality of care [56].

Other variables tested, such as race/skin color at the individual level, neighborhood level variables, and HDI, procedures and rehabilitation at the municipal level lost their association with the outcome during the analysis. It is noteworthy that the only context variable that remained in the final model was the infrastructure of the service highlighting differences from the analysis in which the outcome was oral diseases and / or treatment needs, in which the context was revealed to be a strong determining factor [57].

It is important to highlight that our findings do not provide causal relationships because the database that originated the analysis at the individual level came from a cross-sectional study. Thus, this study may show associations but not the correct time sequence necessary to draw conclusions about causal mechanisms.

Another limitation to be considered is the fact that the dependent variable considers only the type of service used in the last dental appointment, which may be an exception to the type of service usually used. However, when we take into account the randomness of the selection method of survey participants, it is assumed that these situations present a randomized distribution and do not constitute systematic bias [48].

We also emphasize the importance of the study to characterize the context variables for the use of public services; this study notes that better structured public services lead to the increased use of these services by the population. Thus, public policies should ensure funding strategies that provide an increasing in health services coverage, professional training and acquisition of equipment so that services can broaden in scope and consequently ensure the integrality in attendance of its users.

## Conclusion

Much has been discussed about the role of the context in where individuals live and the role of these factors on oral diseases. In this work, we tried to verify whether these factors also contribute to the use of oral health services. It was verified that the individual factors associated with the use of public service were the number of people in the residence, family income and number of teeth requiring treatment. The only contextual factor that influenced the use of public service was the existing infrastructure in the municipalities describing the way these services are organized.

Thus, in addition to acting from an inter institutional perspective for intervention on individual factors, public policies should also mediate the creation of health services more accessible, resolutive and that ensure equity and integrality.

Therefore, investing in oral healthcare services from the creation and expansion of financial incentives, creation and structuring of the facilities for healthcare, organization of patient flow and professional qualification is crucial to the achievement of the required health services in terms of quality and quantity, in order to provide proper care to the population needs.

## References

[1] Instituto Brasileiro de Geografia e Estatística: Um Panorama da Saúde no Brasil: Acesso e utilização dos serviços, condições de saúde e fatores de risco e proteção à saúde—2008. Rio de Janeiro: IBGE; 2010.

[2] Gilbert HG, Duncan RP, Bruce V. Determinants of dental care use in dentate adults: sixmonthly use during a 24-month period in the Florida Dental Care Study. Soc Sci Med. 1998; 47: 727–737. 9690820 10.1016/s0277-9536(98)00148-8

[3] Tennstedt SL, Brambilla DL, Jette AM, McGuire SM. Understanding dental service use by older adults sociobehavioral factors vs. need. J Public Health Dent. 1994; 54: 211–219. 7799295 10.1111/j.1752-7325.1994.tb01217.x

[4] Nguyen L, Häkkinen U. Choices and utilization in dental care Public vs. private dental sectors, and the impact of a two-channel financed health care system. Eur J Health Econom. 2006; 51: 99–106. 10.1007/s10198-006-0344-3 16489469

[5] Luzzi L, Spencer AJ. Public dental service utilization among adults in South Australia. Aust Dent J. 2009; 54: 154–160. 10.1111/j.1834-7819.2009.01109.x 19473158

[6] Brennan DS, Luzzi L, Roberts-Thomson KF. Dental service patterns among private and public adult patients in Australia. BMC Health Serv Res 2008; 8:1 http://www.biomedcentral.com/1472-6963/8/1. 10.1186/1472-6963-8-1 18173837 PMC2246120

[7] Roberts-Thomson KF, Luzzi L, Brennan DS. Social inequality in use of dental services: relief of pain and extractions. Aust N Z J Public Health. 2008; 32: 444–449. 10.1111/j.1753-6405.2008.00277.x 18959548

[8] Hancock M, Calnan M, Manley G. Private or NHS General Dental Service care in the United Kingdom? A study of public perception and experience. J Public Health Med. 1999, 21; 415–420. 11469364 10.1093/pubmed/21.4.415

[9] Ajayi DM, Arigbede AO. Barriers to oral health care utilization in Ibadan, South West Nigeria. Afr Health Sci. 2012; 12: 507–513. 23515140 10.4314/ahs.v12i4.17 PMC3598293

[10] Locker D, Maggirias J, Quiñonez C. Income, dental insurance coverage, and financial barriers to dental care among Canadian adults. J Public Health Dent. 2011; 71: 327–334. 10.1111/j.1752-7325.2011.00277.x 22320291

[11] Slack-Smith L, Lange A, Paley G, O’Grady M, French D, Short L. Oral health and access to dental care: a qualitative investigation among older people in the community. Gerodontology. 2010; 27: 104–113. 10.1111/j.1741-2358.2009.00320.x 19572921

[12] Ribeiro MCSA, Barata RB, Almeida MF, Silva ZP. Sociodemographic profile and utilization patterns of the public health care system (SUS)–PNAD 2003. Cien Saude Colet. 2006; 11: 1011–1022.

[13] Gouveia GC, Souza WV, Luna CF, Souza-Júnior PRB, Szwarcwald CL. Health care users' satisfaction in Brazil, 2003. Cad Saude Publica. 2005, 21(Suppl 1); 109–118. 16463002 10.1590/s0102-311x2005000700012

[14] Pavão ALB, Coeli CM, Lopes CS, Faerstein E, Werneck GL, Chor D. Social determinants of the use of health services among a public university workers. Rev Saude Publica. 2012; 46: 98–103. 22218759 10.1590/s0034-89102012005000002

[15] Martins AMEBL, Haikal DS, Pereira SM, Barreto SM. Routine use of dental services by the elderly in Brazil: the SB Brazil Project. Cad Saude Publica. 2008; 24: 1651–1666. 18670689 10.1590/s0102-311x2008000700020

[16] Matos DL, Lima-Costa MF. Trends in the use of dental services by elderly Brazilians and related socio-demographic factors based on the National Household Survey (1998 and 2003). Cad Saude Publica. 2007; 23: 2740–2748. 17952266 10.1590/s0102-311x2007001100021

[17] Martins AMEBL, Barreto SM, Pordeus IA. Utilization of dental services among the elderly in Brazil. Rev Panam Salud Publica. 2007; 22: 308–316. 18198039 10.1590/s1020-49892007001000003

[18] Matos DL, Lima-Costa MFF, Guerra HL, Marcenes W, Lima-Costa MFF, Guerra HL, Marcenes W. The Bambuí Project: a population-based study of factors associated with regular dental care in adults. Cad Saude Publica. 2001; 17: 661–668. 11395802 10.1590/s0102-311x2001000300020

[19] Camargo MBJ, Dumith SC, Barros AJD. Regular use of dental care services by adults: patterns of utilization and types of services. Cad Saude Publica. 2009; 25: 1894–1906. 19750377 10.1590/s0102-311x2009000900004

[20] Machado LP, Camargo MBJ, Jeronymo JCM, Bastos GAN. Regular use of dental services among adults and older adults in a vulnerable region in Southern Brazil. Rev Saude Publica. 2012; 46: 526–533. 22635038 10.1590/s0034-89102012000300015

[21] Lacerda JT, Simionato EM, Peres KG, Peres MA, Traebert J, Marcenes W. Dental pain as the reason for visiting a dentist in a Brazilian adult population. Rev Saude Publica. 2004; 38: 453–458. 15243677 10.1590/s0034-89102004000300017

[22] Guilera SLVU, Franca BHS, Moyses ST, Moyses SJ. Intermunicipal inequities in access and use of secondary health services in the metropolitan area of Curitiba. Rev Bras Epidemiol. 2014; 17: 654–667. 10.1590/1809-4503201400030007 25272259

[23] Chaves SCL, Soares FF, Rossi TRA, Cangussu MCT, Figueiredo ACL, Cruz DN, Cury PR. Characteristics of the access and utilization of public dental services in medium-sized cities. Cien Saude Colet. 2012; 17:3115–3124. 23175317 10.1590/s1413-81232012001100027

[24] Barros AJD, Bertoldi AD. Inequalities in utilization and access to dental services: a nationwide assessment. Cien Saude Colet. 2002; 7:709–717.

[25] Pinheiro RS, Torres TZG. Access to oral health services between Brazilian States. Cien Saude Colet. 2006; 11: 999–1010.

[26] Manhães ALD, Costa AJL. Access to and utilization of dental services in the State of Rio de Janeiro, Brazil: an exploratory study based on the 1998 National Household Sample Survey. Cad Saude Publica. 2008; 24: 207–218. 18209849 10.1590/s0102-311x2008000100021

[27] Pinto RS, Matos DL, Loyola Filho AI. Characteristics associated with the use of dental services by the adult Brazilian population. Cien Saude Colet. 2012; 17: 532–544. 10.1590/s1413-81232012000200026 22267047

[28] Pinto RS, Abreu MHNG, Vargas AMD. Comparing adult users of public and private dental services in the state of Minas Gerais, Brazil. BMC Oral Health. 2014; 14: 100 10.1186/1472-6831-14-100 25099268 PMC4130879

[29] Miranda CDBC, Peres MA. Determinants of dental services utilization by adults: a population-based study in Florianópolis, Santa Catarina State, Brazil. Cad. Saude Publica. 2013; 29: 2319–2332. 10.1590/0102-311x00139912 24233046

[30] Instituto Brasileiro de Geografia e Estatística. Estimativas de população para 1° de julho de 2012. http://www.ibge.gov.br/home/estatistica/populacao/estimativa2012/estimativa_tcu.shtm.

[31] Ministério da Saúde. DATASUS: CNES—RECURSOS HUMANOS—PROFISSIONAIS—INDIVÍDUOS—SEGUNDO CBO 2002—MINAS GERAIS. http://tabnet.datasus.gov.br/cgi/tabcgi.exe?cnes/cnv/prid02mg.def.

[32] Agencia Nacional de Saúde Suplementar. Dados e indicadores do setor: Beneficiários de planos privados de saúde. http://www.ans.gov.br/materiais-para-pesquisas/perfil-do-setor/dados-e-indicadores-do-setor.

[33] Secretaria de Estado de Saúde de Minas Gerais. SB Minas Gerais: Pesquisa das condições de saúde bucal da população mineira—Resultados principais. Belo Horizonte: Editora Autêntica; 2013.

[34] Ministério da Saúde. DATASUS: PRODUÇÃO AMBULATORIAL DO SUS—MINAS GERAIS—POR LOCAL DE ATENDIMENTO. http://tabnet.datasus.gov.br/cgi/deftohtm.exe?sia/cnv/qamg.def

[35] Ministério da Saúde. Sala de Apoio à Gestão Estratégica. http://189.28.128.178/sage/

[36] Hair JF Jr, Black WC, Babin BJ, Anderson RE, Tatham RL. Multivariate Data Analysis. 6th ed. New Jersey: Pearson Prentice Hall; 2006.

[37] Programa das Nações Unidas para o Desenvolvimento. Atlas do desenvolvimento humanos dos municípios– 2013. http://www.atlasbrasil.org.br/2013/

[38] Minayo MCS, Hartz ZMA, Buss PM. Quality of life and health: a necessary debate. Cien Saude Colet. 2000; 5: 7–18. 10.1590/S1413-81232000000100002

[39] Instituto Brasileiro de Geografia e Estatística. Censo Demográfico 2010: Características da população e dos domicílios Resultados do universo. Rio de Janeiro: IBGE; 2011 http://www.ibge.gov.br/english/estatistica/populacao/censo2010/caracteristicas_da_populacao/resultados_do_universo.pdf

[40] Instituto Brasileiro de Geografia e Estatística. Base de informações do Censo Demográfico 2010: Resultados do Universo por setor censitário—Documentação do Arquivo. Rio de Janeiro: IBGE 2011 http://www.ipea.gov.br/redeipea/images/pdfs/base_de_informacoess_por_setor_censitario_universo_censo_2010.pdf

[41] Roncalli AG, Silva NN, Nascimento AC, Freitas CHSM, Casotti E, Peres KG et al Relevant methodological issues from the SBBrasil 2010 Project for national health surveys. Cad. Saúde Pública, 2012; 28, supl 10.1590/S0102-311X2012001300006 22714967

[42] Snijders TAB, Bosker RJ. Multilevel analysis: an introduction to basic and advanced multilevel modeling. London: Sage Publications; 2003.

[43] Narvai PC, Antunes JLF, Moysés SJ, Frazão P, Peres MA, Peres KG, et al Scientifi c validity of epidemiological knowledge based on data from the Brazilian Oral Health Survey (SB Brazil 2003). Cad Saude Publica. 2010; 26: 647–70. 20512200 10.1590/s0102-311x2010000400002

[44] Sheiham A, Alexander D, Cohen L, Marinho V, Moysés S, Petersen PE, et al Global oral health inequalities: task group—implementation and delivery of oral health strategies. Adv Dent Res. 2011; 23: 259–67. 10.1177/0022034511402084 21490238

[45] Peres KG, Peres MA, Boing AF, Bertoldi AD, Bastos JL, Barros AJD. Redução das desigualdades na utilização de serviços odontológicos no Brasil entre 1998 e 2008. Rev Saude Publica. 2012; 46: 250–8. 22437856 10.1590/s0034-89102012000200007

[46] Bós AMG, Bós AJG. Determinants of elders’ choice between private and public health care providers. Rev Saude Publica. 2004, 38:113–120 14963550 10.1590/s0034-89102004000100016

[47] Andersen AS, Laake P. A causal model for physician utilization: analysis of Norwegian data. Med Care. 1983; 21: 266–278. 6834905 10.1097/00005650-198303000-00002

[48] Mendoza-Sassi R, Béria JU. Health services utilization: a systematic review of related factors. Cad Saude Publica. 2001; 17: 819–832. 11514863 10.1590/s0102-311x2001000400016

[49] Anikeeva O, Brennan DS, Teusner DN. Household income modifies the association of insurance and dental visiting. BMC Health Services Research. 2013; 13: 432 http://www.biomedcentral.com/1472-6963/13/432. 10.1186/1472-6963-13-432 24153023 PMC4015366

[50] Wamala S, Merlo J, Boström G: Inequity in access to dental care services explains current socioeconomic disparities in oral health: The Swedish National Surveys of Public Health 2004–2005. J Epidemiol Community Health 2006, 60:1027–1033. 17108297 10.1136/jech.2006.046896 PMC2465506

[51] World Health Organization (WHO): Country cooperation strategy at a glance. Brazil: Geneva; 2009. http://apps.who.int/iris/bitstream/10665/70263/1/WHO_DGR_CCO_09.03_Brazil_eng.pdf?ua=1.

[52] Guiotoku SK, Moysés ST, Moysés SJ, França BHS, Bisinelli JC. Racial inequity in oral health in Brazil. Rev Panam Salud Publica. 2012; 31: 135–41. 22522876 10.1590/s1020-49892012000200007

[53] Cohen-Carneiro F, Souza-Santos R, Pontes DG, Salino AV, Rebelo MAB. Provision and utilization of dental services in Amazonas State, Brazil: a case study in a riverine population in Coari Municipality. Cad Saude Publica. 2009; 25: 1827–1838. 19649424 10.1590/s0102-311x2009000800019

[54] Macinko J, Lima-Costa MF. Horizontal equity in health care utilization in Brazil, 1998–2008. Int J Equity Health. 2012; 11(33). http://www.equityhealthj.com/content/11/1/33. 10.1186/1475-9276-11-33 PMC3444440 22720869

[55] FDI World Dental Federation. Oral Health Worldwide: A report by FDI World Dental Federation. Geneva; 2014. http://www.worldoralhealthday.com/wp-content/uploads/2014/03/FDIWhitePaper_OralHealthWorldwide.pdf

[56] Assis MMA, Jesus WLA. Acesso aos serviços de saúde: abordagens, conceitos, políticas e modelo de análise. Cien Saude Colet. 2012; 17: 2865–2875. 23175292 10.1590/s1413-81232012001100002

[57] Roncalli AG, Tsakos G, Sheiham A, Souza GC, Watt RG. Social determinants of dental treatment needs in Brazilian adults. BMC Public Health. 2014; 14: 1097 10.1186/1471-2458-14-1097 25339315 PMC4287338

